# PD-L1-related IncRNAs are associated with malignant characteristics and immune microenvironment in glioma

**DOI:** 10.18632/aging.205120

**Published:** 2023-10-12

**Authors:** Zhiwei Xia, Ruxin Tu, Fangkun Liu, Hao Zhang, Ziyu Dai, Zeyu Wang, Peng Luo, Shiqing He, Gelei Xiao, Jie Feng, Quan Cheng

**Affiliations:** 1 Department of Neurology, Hunan Aerospace Hospital, Changsha 410205, Hunan, P.R. China; 2 Department of Neurology, Xiangya Hospital, Central South University, Changsha 410008, Hunan Province, P.R. China; 3 Department of Neurosurgery, Xiangya Hospital, Central South University, Changsha 410008, Hunan Province, P.R. China; 4 National Clinical Research Center for Geriatric Disorders, Xiangya Hospital, Central South University, Changsha 410008, Hunan, P.R. China; 5 Department of Neurosurgery, The Second Affiliated Hospital, Chongqing Medical University, Chongqing 400010, P.R. China; 6 MRC Centre for Regenerative Medicine, Institute for Regeneration and Repair, University of Edinburgh, Little France, Edinburgh, EH16 4UU, UK; 7 Department of Oncology, Zhujiang Hospital, Southern Medical University, Guangzhou 510280, P.R. China; 8 Department of Neurosurgery, Affiliated Nanhua Hospital, Hengyang Medical College, University of South China, Hengyang 421001, Hunan, P.R. China; 9 Hunan Clinical Research Center for Cerebrovascular Disease, Changsha 410008, Hunan Province, P.R. China

**Keywords:** glioma, PD-L1, LINC01271, risk score, prognosis

## Abstract

Background: The expression of long non-coding RNA (lncRNA) can function as diagnostic and therapeutic biomarker for tumors. This research explores the role of PD-L1-related lncRNAs in affecting malignant characteristics and the immune microenvironment of glioma.

Methods: Downloading gene expression profiles and clinicopathological information of glioma from TCGA and CGGA databases, 6 PD-L1-related lncRNAs were identified through correlation analysis, Cox and LASSO regression analysis, establishing the risk score model based on them. Bioinformatics analysis and cell experiments *in vitro* were adopted to verify the effects of LINC01271 on glioma.

Results: Risk scores based on 6 PD-L1-related lncRNAs (AL355974.3, LINC01271, AC011899.3, MIR4500HG, LINC02594, AL357055.3) can reflect malignant characteristics and immunotherapy response of glioma. Patients with high LINC01271 expression had a worse prognosis, a higher abundance of M1 subtype macrophages in the immune microenvironment, and a higher degree of tumor malignancy. Experiments *in vitro* confirmed its positive regulatory effect on the proliferation and migration of glioma cells.

Conclusions: The risk score model based on 6 PD-L1-related lncRNAs can reflect the malignant characteristics and prognosis of glioma. LINC01271 can independently be used as a new target for prognosis evaluation and therapy.

## INTRODUCTION

Glioma is of great concern because of its high fatality rate. According to the degree of malignancies, lower-grade gliomas (LGG) are classified as World Health Organization (WHO) II and III, including diffuse low-grade and intermediate-grade gliomas. In contrast, glioblastomas (GBM) belong to WHO grade IV [1], with the highest incidence and lowest survival [2]. Surgical resection is mainly applied to treat glioma, combined with radiotherapy, chemotherapy, and other comprehensive methods [3]. Nevertheless, the therapies are inefficacious due to the infiltration and invasion of glioma. The median overall survival (OS) of GBM patients is only 12-18 months after diagnosis, even under the optimal treatment [4]. For decades, researchers have actively explored glioma's pathological features to discover biomarkers for early diagnosis, providing targets for gene therapy and immunotherapy [3–6], aiming to provide better health care and personalized medicine for glioma patients.

Immunotherapy has greatly advanced the treatment of malignant tumors, with immune checkpoint inhibitors (ICIs) playing a crucial role. Classical immune checkpoints include programmed cell death protein 1 (PD-1), PD-L1 and cytotoxic T lymphocyte antigen 4 (CTLA-4). The ICIs can inhibit the interaction between the ligand and immunosuppressive receptors, thereby preventing immune evasion [7]. Nowadays, ICIs have shown significant advantages in lung cancer and breast cancer [5, 8]. This study starts with PD-L1-related lncRNAs, elucidating the immunization characteristics of glioma and their prognostic implications.

In the last decade, lncRNAs have gained recognition for their significant biological effects. They are defined as RNA molecules longer than 200 nucleotides that cannot encode proteins [6]. They play a critical role in many life activities such as epigenetics, cell cycle, and differentiation. Their expression is strictly regulated under physiological conditions [9]. LncRNAs’ function as biomarkers or therapeutic targets for tumors has received extensive attention [10–13]. Lu et al. found that N6-methyladenosine-related non-coding RNAs showed excellent performance in predicting prognosis and immunotherapy response in bladder cancer [14]. Lu et al. proved the potential of lncRNAs as prognostic markers and personalized therapeutic targets for ovarian cancer treatment [15, 16]. The abnormal expression of lncRNAs is associated with glioma occurrence, progression, invasiveness, and recurrence [17–20], through diverse molecular mechanisms that directly or indirectly regulate gene expression [21, 22]. However, there is a paucity of studies investigating PD-L1-related lncRNAs in gliomas.

**Figure 1:**
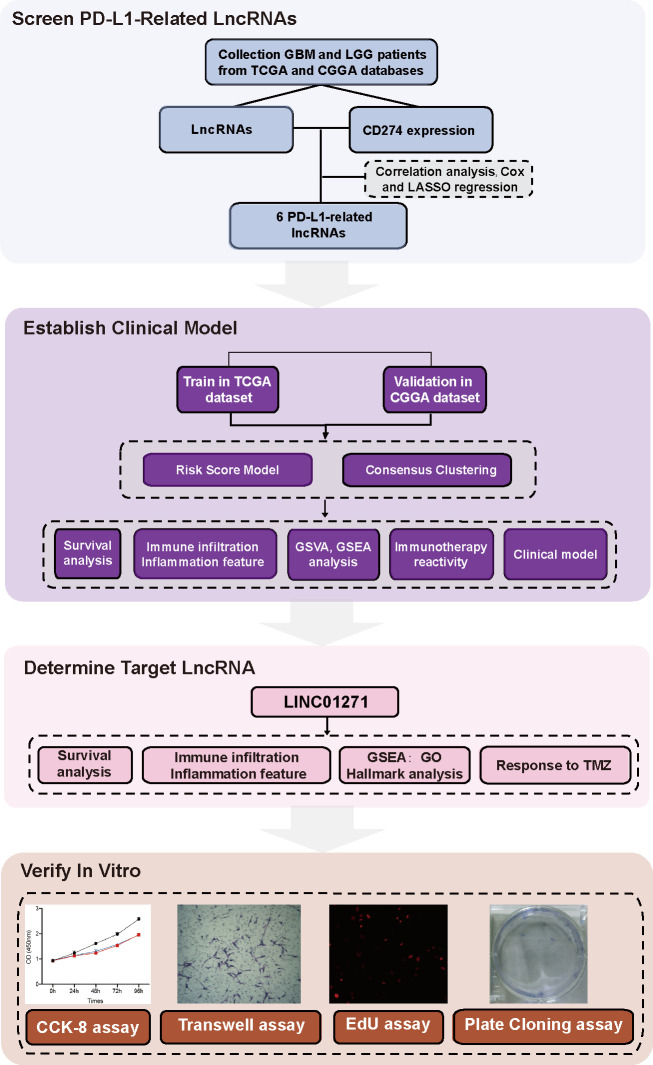
Flow chart.

Herein, for the first time, we established a risk score model containing 6 PD-L1-related lncRNAs to reflect individual heterogeneity of gliomas and provide a tool for prognostic stratification. LINC01271 was chosen as the target lncRNA to validate its predictive efficacy on the clinical and pathological features, and prognosis of glioma (Figure 1 Flow Chart).

## RESULTS

### Identification of lncRNAs related to PD-L1

**Figure 2:**
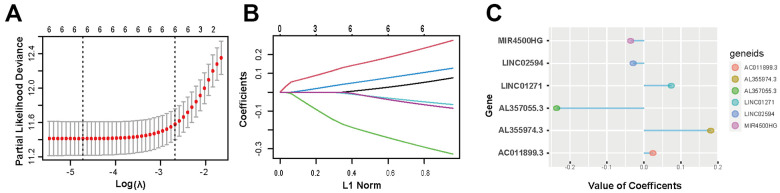
**Screening of 6 PD-L1-related lncRNAs.** (**A**, **B**) LASSO regression analysis is employed to determine the optimal penalty coefficient (λ). (**C**) Regression coefficients of 6 PD-L1-related lncRNAs.

The genetic information, RNA transcriptome data, and related clinical data of glioma patient samples were acquired from the TCGA and CGGA database. PD-L1 is encoded by gene *CD274*. Through correlation analysis and univariate logistic regression analysis, 23 lncRNAs were selected from 13895 lncRNAs associated with *CD274* expression. Then multivariate cox analysis was used to figure out lncRNAs independently related to *CD274*. With p<0.05 as the standard, 6 lncRNAs (AL355974.3, LINC01271, AC011899.3, MIR4500HG, LINC02594, AL357055.3) were screened. Finally, further dimension reduction and model construction were performed through LASSO analysis (Figure 2A–2C and Supplementary Table 1).

### Risk score model can predict the prognosis of glioma patients

We observed the effects of the 6 PD-L1-related lncRNAs on the prognosis of glioma in TCGA database. Kaplan-Meier survival curve analysis showed AL355974.3 (HR=4.06, p<0.001), LINC01271 (HR=3.51, p<0.001), and AC011899. 3 (HR=2.66, p<0.001) were significantly related to a poorer prognosis; MIR4500HG (HR=0.39, p<0.001), LINC02594 (HR=0.48, p<0.001), and AL357055.3 (HR=0.28, p<0.001) were relevant to a better prognosis (Supplementary Figure 1). The findings suggest that using a prediction model based on these 6 lncRNAs can enhance the prognostic accuracy of glioma.

We established a risk score model based on the 6 PD-L1-related lncRNAs. The correlation analysis revealed a positive association between AL355974.3, LINC01271, and AC011899.3 with the risk score, whereas MIR4500HG, LINC02594 and AL357055.3 exhibited an inverse correlation (Supplementary Figure 2).

**Figure 3:**
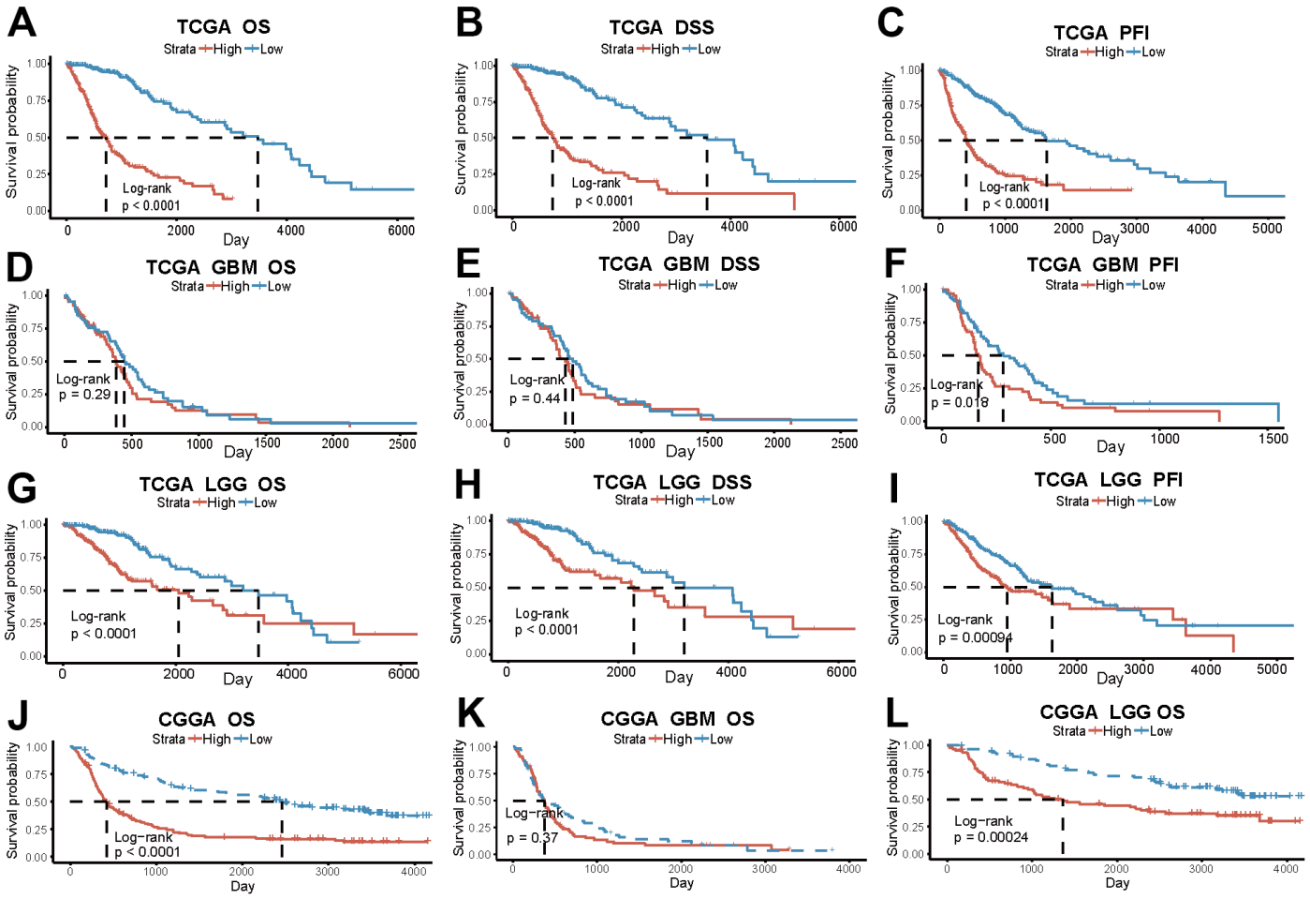
**The risk score based on PD-L1-related lncRNAs indicates glioma patients’ prognosis.** (**A**–**C**) OS, DSS, PFI in the high and low-risk group of all glioma patients in TCGA. (**D**–**F**) OS, DSS, PFI of GBM patients in TCGA. (**G**–**I**) OS, DSS, PFI of LGG patients in TCGA. (**J**–**L**) OS of all patients, GBM and LGG patients in CGGA.

The prognostic outcome of glioma is intricately linked to the risk score. The survival analysis of the TCGA data revealed that, among all glioma patients, the low-risk score group exhibited significantly superior prognosis compared to the high-risk score group (p<0.0001) (Figure 3A–3C). However, in terms of tumor pathological classification, only the PFI shows a significant difference among GBM patients with varying risk scores (p=0.018). In contrast, the three prognostic indicators of LGG patients are all significant differences (OS, DSS: p<0.0001, PFI: p=0.00094) (Figure 3D–3I). The survival analysis in the CGGA validation dataset, which contains 306 patients, yields similar results. Significantly different OS between two risk groups exists among both all glioma and separate LGG patients (OS_ALL_: P <0.0001; OS _LGG_: P = 0.00024), but not in GBM subgroup (p=0.37) (Figure 3J–3L).

To further explore the prognostic efficiency of PD-L1-related lncRNAs, we use consensus cluster analysis to group samples, a clear distinction between the groups was observed when k=2 (Supplementary Figure 3A–3C). Cluster1 and Cluster2 can be distinguished by principal component analysis (PCA), proving the clustering rationality (Supplementary Figure 3D, 3E). The risk score for Cluster1 generated by cluster analysis is significantly higher than that of Cluster2 (p<2.2e-16) (Supplementary Figure 3F). Survival analysis using TCGA data showed worse prognosis of Cluster 1 among all glioma patients and LGG subgroup. But in GBM subgroup, only PFI had a statistical difference (Supplementary Figure 3G–3O). In the CGGA database, poorer prognosis of Cluster 1 patients was showed both in the overall glioma patients and in the subgroup analysis (Supplementary Figure 3P–3R).

These results suggest that the risk score model, which is based on 6 PD-L1-related lncRNAs, exhibits favorable predictive efficacy in glioma patient prognosis, especially in the LGG patient group.

### Risk score is correlated with the clinical, pathological, and genetic characteristics of glioma

**Figure 4:**
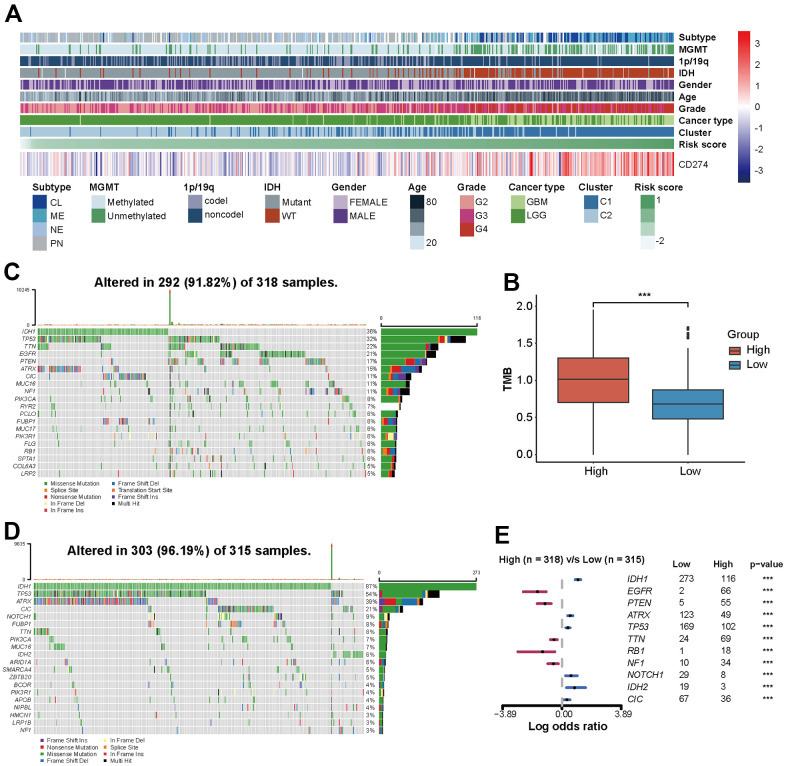
**The risk score of PD-L1-related lncRNAs reflects the clinical, pathological, and genetic characteristics of glioma.** (**A**) Heatmap displaying risk scores, clinical information, and pathological features of glioma patients. (**B**) Analysis of tumor mutation burden (TMB). (**C**, **D**) 20 mutation sites in high-risk and low-risk groups. (**E**) Risk score-related somatic mutations.

The association between risk scores and clinical features of glioma was investigated. 672 patients enrolled from TCGA database were categorized into two groups based on the median risk score, with high and low expression groups identified. Patients with high-risk score tended to be older and more frequently diagnosed with GBM, which exhibited IDH wild type, non-codel of 1p/19q, and unmethylated MGMT pathological features (Table 1). Heatmap and box plots provide a more intuitive and accurate representation, patients with high-risk scores had higher levels of *CD274* expression and more malignant glioma pathologic features, regardless of gender (Figure 4A and Supplementary Figure 4A–4H). Among GBM and LGG subgroups, IDH wild type was more common in high-risk groups, there was no significant difference in MGMT and 1p/19q status (Supplementary Figure 4I–4L).

**Table 1:** Clinical characteristics of patients with high and low risk score.

**Characteristic**	**N**	**High, N=336^1^**	**Low, N=336^1^**	**p-value^2^**
**Age**	672	54(42,63)	38(30,48)	<0.001
**Gender**	672			>0.9
Female		143(43%)	142(42%)	
Male		193(57%)	194(58%)	
**Cancer**	672			<0.001
GBM		143(43%)	7(2.1%)	
LGG		193(57%)	329(98%)	
**IDH**	662			<0.001
Mutant		122(37%)	312(94%)	
WT		207(63%)	21(6.3%)	
**1p/19q**	668			<0.001
codel		50(15%)	121(36%)	
non-codel		282(85%)	215(64%)	
**MGMT**	635			<0.001
Methylated		184(61%)	294(88%)	
Unmethylated		117(39%)	40(12%)	

^1^Median(IQR); n(%).

^2^Wilcoxon rank sum test; Pearson’s Chi-squared test.

Genomic heterogeneity leads to prognostic differences in tumors. Patients with higher tumor mutation burden (TMB) have higher risk scores (p<0.001, Figure 4B). In the TCGA set, 292 (91.82%) of the 318 patients with high-risk score and 303 (96.19%) of the 315 patients with low-risk score had somatic mutations (Figure 4C, 4D). Genome variation analysis shows that low-risk patients have higher mutation frequency of IDH1, ATRX, TP53, NOTCH1, IDH2, and CIC (p <0.001), while EGFR, PTEN, TTN, RB1, and NF1 gene mutation frequency is higher in a high-risk group (p<0.001) (Figure 4E).

### Risk score correlates with the immune inflammatory microenvironment and immunotherapy responsiveness of glioma

Immune and inflammatory cells play a crucial role in the composition of tumor tissue, with the tumor immune microenvironment (TIME) exerting significant influence on both disease progression and response to treatment. We use GSVA to identify the immune infiltration and inflammation profiles associated with characteristic lncRNAs and draw correlation maps and heatmaps.

**Figure 5:**
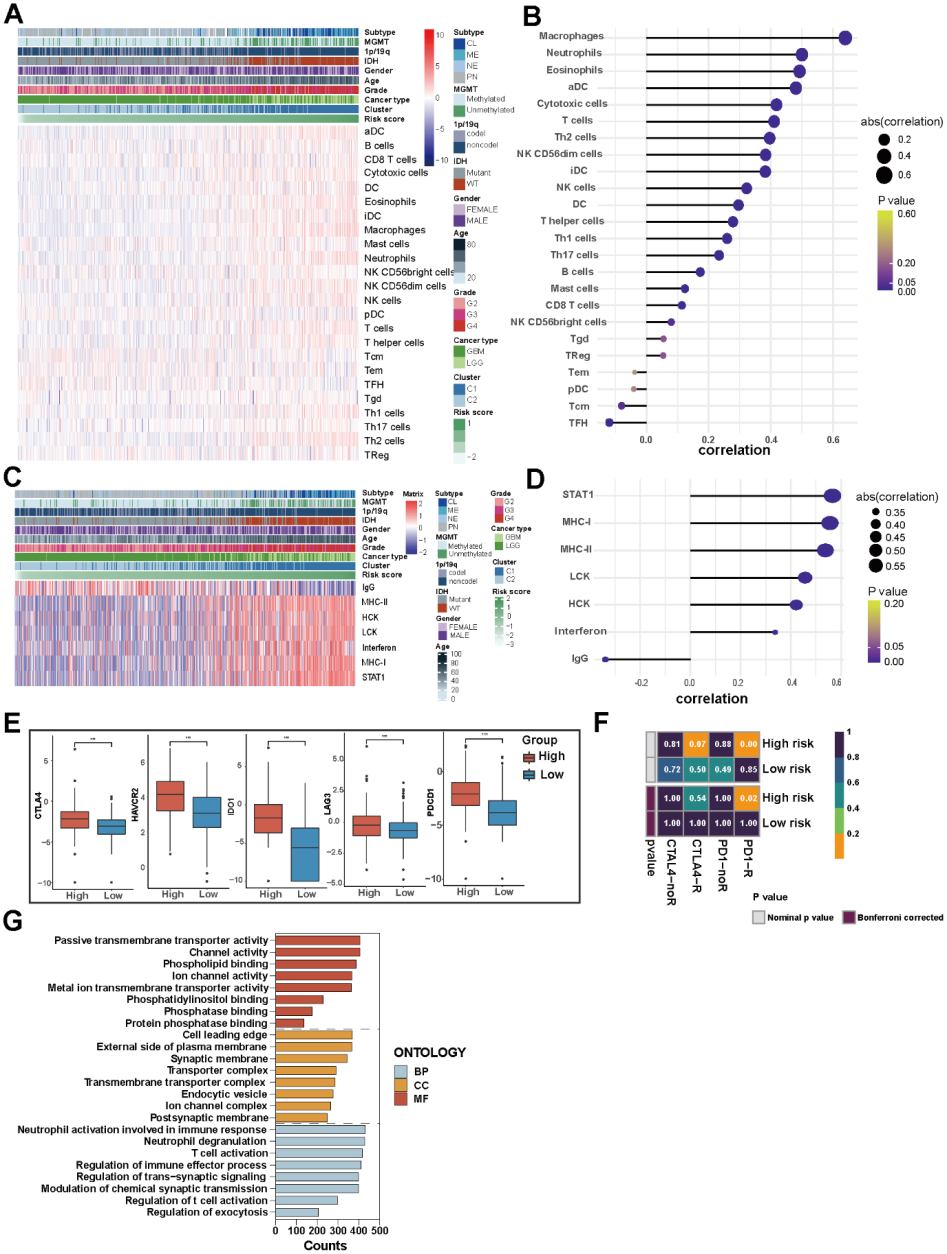
**Immune and inflammatory characteristics of glioma and the biological mechanism of PD-L1-related lncRNAs.** (**A**) Heatmap displaying the characteristics of immune cells panel. (**B**) The correlation between immune cell infiltration and risk score. (**C**) Heatmap displaying the characteristics of inflammation panel. (**D**) The correlation between inflammation characteristics and risk score. (**E**) 5 immune checkpoints’ expression in high-risk and low-risk groups in TCGA. (**F**) Patients’ predicted response to anti-CTLA-4 and anti-PD-1 therapy. (**G**) GO analysis of PD-L1-related lncRNAs.

To clarify whether PD-L1-related lncRNAs affect prognosis by influencing the tumor immunity and inflammation, we compared the degree of immune cell infiltration. Patients in the high-risk group exhibited a greater diversity of immune cell infiltration compared to those in the low-risk group. In TCGA data, a significant increase in macrophages (M0, M1), neutrophils, CD8^+^ T cells, T follicular helper cells and eosinophils within the high-risk score group was observed (p<0.001). While patients in the low-risk score group exhibited a marked elevation in monocytes (P<0.001) (Figure 5A, 5B and Supplementary Figure 5A). Consistently, in the CGGA set, macrophages had a higher abundance and relevance of risk score in the high-risk score group and Cluster 1 (Supplementary Figure 5B, 5C).

In TCGA set, high-risk group and Cluster 1 were associated with higher expression of MHC-I, MHC-II, LCK, HCK, interferon and particularly STAT1 among inflammation-related molecules. Meanwhile, IgG showed increased expression in low-risk group and Cluster 2 (Figure 5C, 5D). The results of the CGGA dataset verification reveal slight variations in the relevance degrees of 7 molecules, with MHC-1 exhibiting the most robust positive association with high-risk scores (Supplementary Figure 5D, 5E). This suggests that PD-L1-related lncRNAs regulate lymphocyte activation, activation of antigen-presenting cells and interferon signalling in gliomas.

The growth and progression of cancer are in connection with immunosuppression. ICIs have been considered revolutionary immunotherapy for a variety of tumors, including glioma; the tumor microenvironment influences the response of the tumor to immune checkpoint inhibitor therapies. We analyzed the expression of five common immune checkpoints (CTLA-4, HAVCR2, IDO1, LAG3, PDCD1) in patients, and found that people with high-risk scores had higher expression of these five immune checkpoints (P<0.001, Figure 5E). We further forecasted the patient’s response to anti-PD-1 and anti-CTLA-4 therapies. We observed a higher likelihood of sensitivity to anti-PD-1 treatment among patients in the high-risk group (p<0.05), while the response predicted to anti-CTAL-4 treatments of the two groups is similar (Figure 5F). Risk scores based on PD-L1-related lncRNAs can predict response to immunotherapy in glioma patients and thus guide personalized medicine.

For a profound understanding of the potential biological mechanisms of PD-L1-related lncRNAs, GSEA analysis was conducted. The results revealed that the biological processes of PD-L1-related lncRNAs were enriched in neutrophil activation involved in immune response, neutrophil degranulation, and T cell activation. Cell components were mainly concentrated at the leading edge of the cell, on the outer side of both the plasma membrane and synaptic membrane. The molecular functions were focused on passive transmembrane transporter activity, channel activity and phospholipid binding (Figure 5G).

### Clinical models based on PD-L1-related lncRNAs and clinical characteristics can predict the prognosis of glioma patients

**Figure 6:**
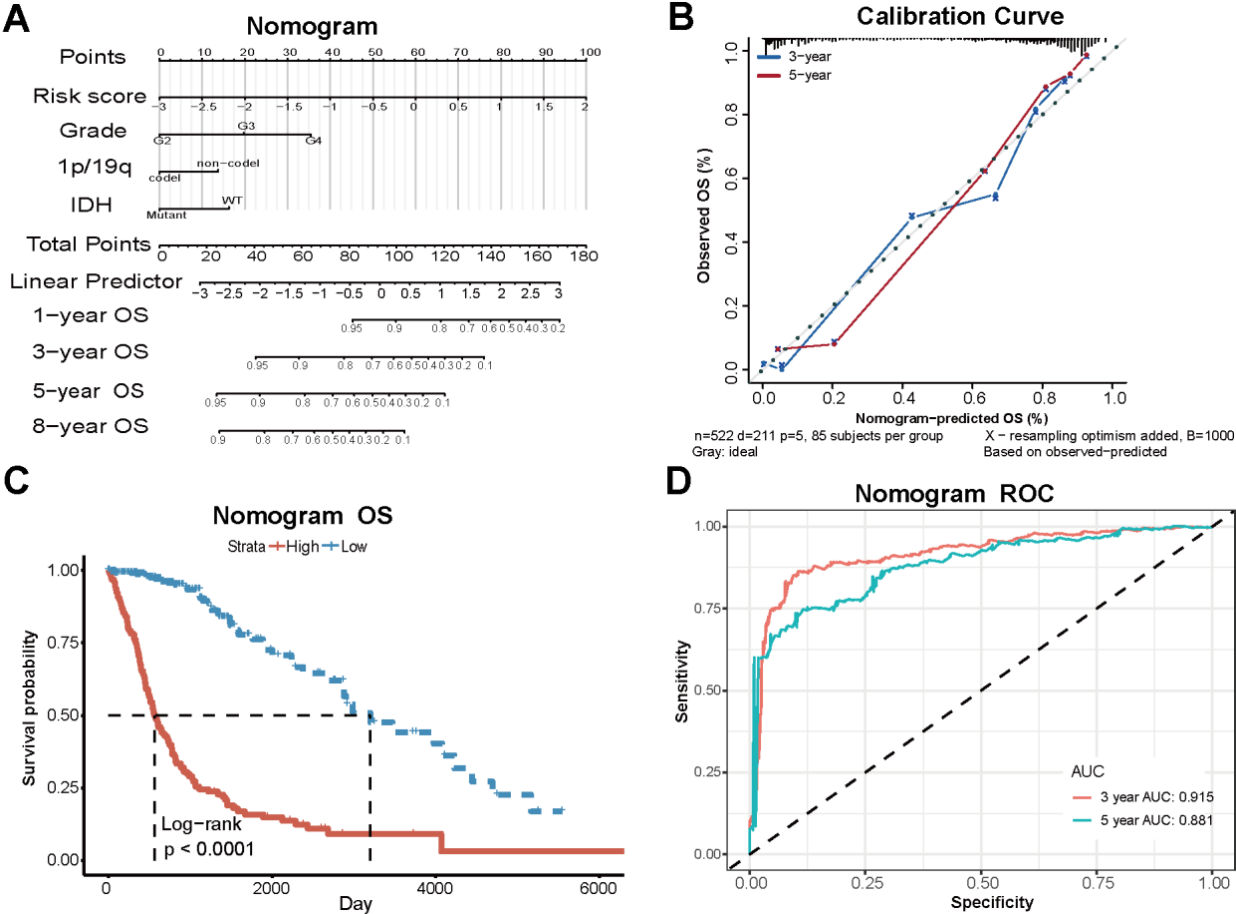
**Clinical models based on PD-L1-related lncRNAs and clinical characteristics.** (**A**) The nomogram combining risk scores and clinical case characteristics according to TCGA data. (**B**) The calibration curves showing the predicted and actual observed OS rates. (**C**) Survival analysis of new high-risk group and low-risk group. (**D**) The ROC curves and AUC values of the nomogram.

Based on the TCGA dataset, we developed a clinical model that combines risk scores and clinical case characteristics, and created a nomogram (Figure 6A). The model’s predicted 3-year and 5-year OS closely align with the observed outcomes (Figure 6B), suggesting that the model has a specific predictive value. Taking account of the nomogram, the patients were divided into two new risk groups. Survival analysis showed a conspicuously different prognosis in the two groups (P<0.0001) (Figure 6C). The 3-year AUC under the ROC curve is 0.915, while the 5-year AUC is 0.881. The robust specificity and sensitivity of our model demonstrate its superior predictive power compared to using risk score alone when incorporating clinicopathological characteristics (Figure 6D).

### LINC01271 is significantly associated with features of glioma

According to previous studies, Diermeier’s team found that lncRNA Mammary Tumor-associated RNA 25 (MaTAR25) contributed to the malignancy of breast tumor cells *in vitro* and *in vivo*. LINC01271 was identified as a homologous human lncRNA of MaTAR25, and they proved that increased LINC01271 expression was relevant to poor prognosis and metastasis [23, 24]. The role of LINC01271 in gliomas has not been reported, so we selected LINC01271 as the target to investigate its impact on glioma development and prognosis.

**Figure 7:**
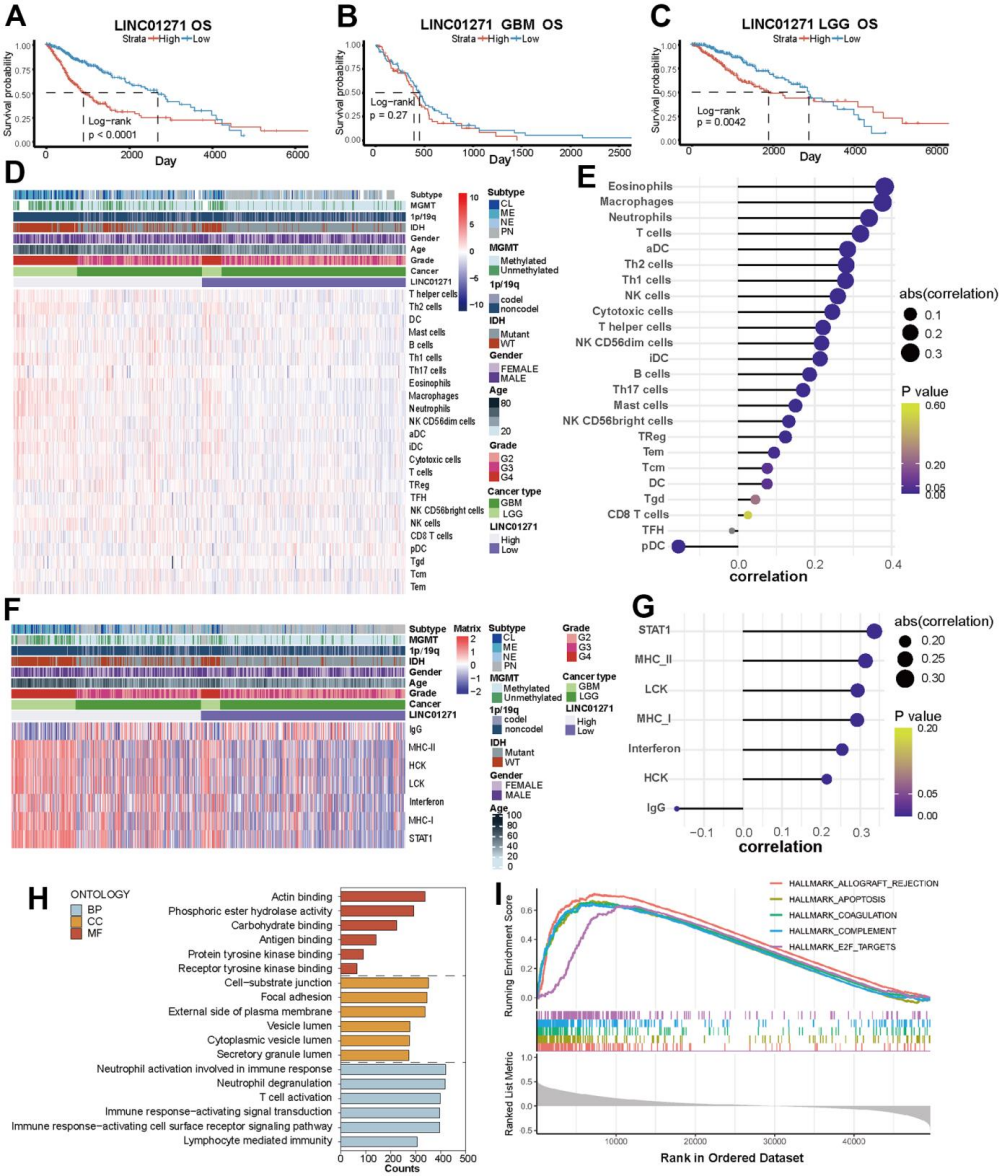
**Correlations between LINC01271 expression and glioma prognosis, immune and inflammatory features.** (**A**–**C**) OS of glioma patients with high and low LINC01271 expression in TCGA. (**D**) Heatmap displaying the characteristics of immune cells panel. (**E**) Correlation between immune cell infiltration and LINC01271 level. (**F**) Heatmap displaying the characteristics of inflammation panel. (**G**) Correlation between inflammation characteristics and LINC01271 level. (**H**) GO analysis of LINC01271 related genes. (**I**) Hallmark analysis of LINC01271 related genes.

The glioma patients in two data sets were separated into two different groups according to LINC01271 expression level. In the TCGA dataset, the overall OS, DSS, and PFI of patients with high LINC01271 expression were worse than another group (p<0.0001) (Figure 7A and Supplementary Figure 6A, 6D), but from the perspective of cancer type, the prognostic difference was only reflected in LGG (LGG: OS p=0.0042, DSS p =0.0083, PFI p<0.0001; GBM: OS p=0.27, DSS p=0.33, PFI p=0.46) (Figure 7B, 7C and Supplementary Figure 6B, 6C, 6E, 6F), which is in line with the results in the CGGA set (Supplementary Figure 6G–6I).

Then we studied the correlation between LINC01271 expression and the characteristics of immunity, inflammation, and clinicopathology. The results showed more infiltrates of M1 subtype macrophages, CD8^+^ T cells and T follicular helper cells in the high LINC01271 expression group (Figure 7D and Supplementary Figure 6J), and the infiltration degree of eosinophils, macrophages, neutrophils and T cells was positively correlated with the LINC01271 expression, while pDC was negatively correlated with the expression of LINC01271 (Figure 7E). Low LINC01271 expression group showed more infiltration of NK cells and Mast cells (Supplementary Figure 6J). The same analysis was conducted on CGGA database samples, yielding a heatmap with comparable findings (Supplementary Figure 6K).

Inflammatory characteristics analysis shows that STAT1, MHC-II, LCK, MHC-I, interferon, and HCK are associated with high LINC01271 expression. In contrast, patients with low LINC01271 expression have higher IgG expression (Figure 7F, 7G). These findings are consistent with the previous analysis results based on the 6 PD-L1-related lncRNAs.

As for clinicopathological characteristics, we found that CL, ME subtype, MGMT non-methylation, 1p19q non-codel, IDH wild type, higher age, higher WHO grade, and higher GBM patients’ proportion are associated with high LINC01271 expression, which illustrates that LINC01271 is a risk factor for prognosis (Figure 7D, 7F).

GSEA analysis revealed that LINC01271 mainly participated in the same biological process as the 6 PD-L1-related lncRNAs model, but concentrated on different cellular components, including the cell-substrate junction, focal adhesion, external side of the plasma membrane and others, and were related to molecular functions like acting binding and phosphoric ester hydrolase activity (Figure 7H). The expression of LINC01271 is also in connection with some hallmark gene sets, such as APOPTOSIS, COAGULATION, COMPLEMENT, and E2F TARGETS (Figure 7I), suggesting the immune pathways related to characteristic genes, and perhaps related to the mechanism of LINC01271’s biological role in glioma.

We further conducted a prediction of the patients’ sensitivity to TMZ treatment, which is currently one of the most commonly used chemotherapy drugs for glioma. With data derived from the GDSC database, we built a predictive model and estimated the half-maximal inhibitory concentration (IC50) value of TMZ in each patient. Unfortunately, there was no statistical difference in IC50 when grouping according to LINC01271 expression (Supplementary Figure 6L). Finally, we compared the LINC01271 expression in tumor and normal tissues using the TCGA database, but no statistically significant difference was observed (Supplementary Figure 6M).

### Knockout of LNC01271 effectively suppressed the proliferation and migration of glioma cells

**Figure 8:**
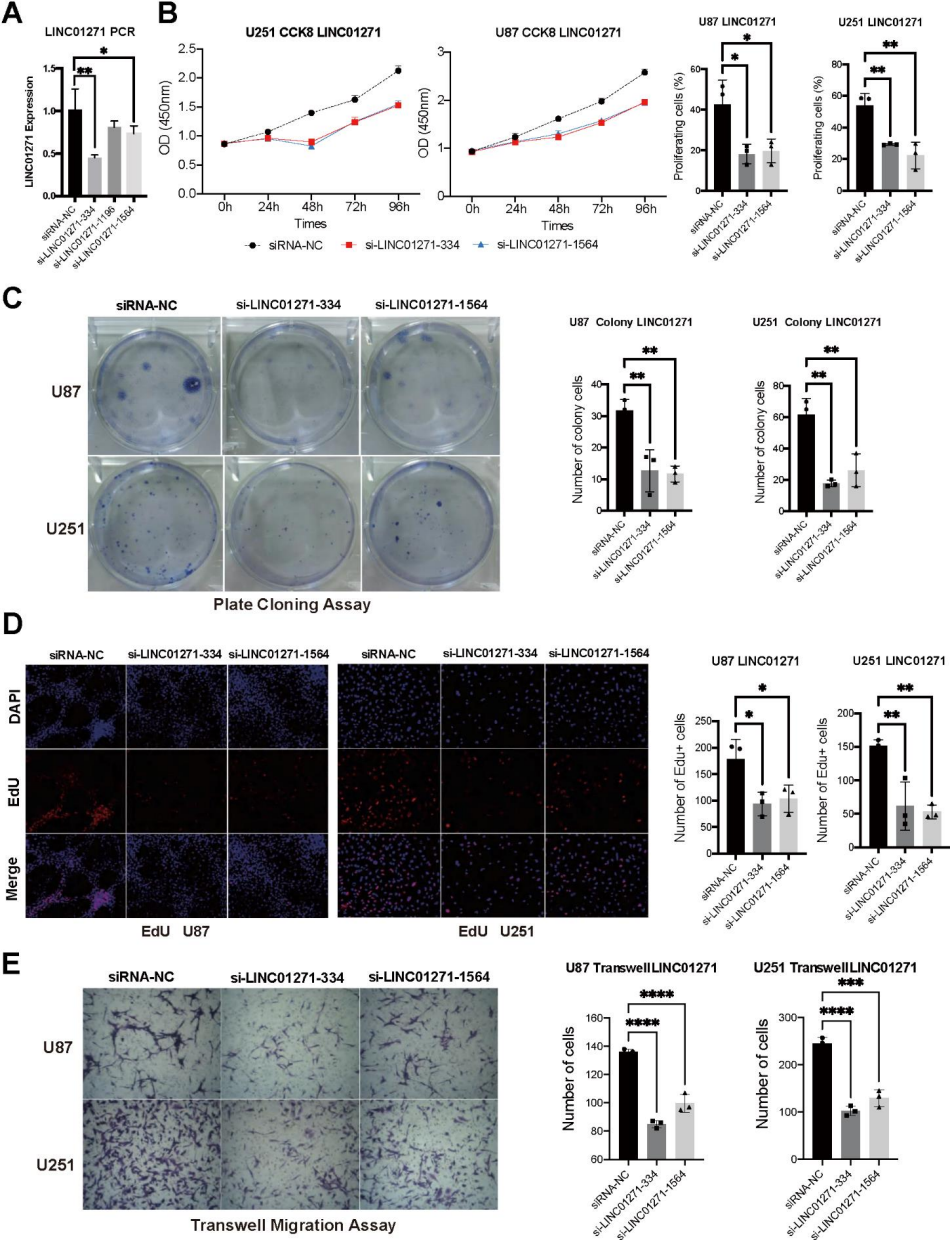
**Validation of LINC01271 in positively regulating glioma cell proliferation and migration *in vitro*.** (**A**) *LINC01271* expression after silencing. (**B**–**D**) CCK-8, plate clone assay, EdU verified the proliferation of glioma cells after LINC01271 silencing. (**E**) Glioma cell migration after *LINC01271* silencing.

We silence *LINC01271* gene expression in U251 and U87 to detect proliferation and migration. RT-qPCR confirmed the silencing efficiency of siRNA, si-LINC01271-334 and si-LINC01271-1564 significantly reduced the expression of *LINC01271*, si-LINC01271-1196 also had the same trend (Figure 8A). CCK-8 assay, plate cloning assay, and Edu assay showed that tumor cell proliferation and colony formation were inhibited after silencing *LINC01271* (Figure 8B–8D). Transwell migration assay suggested inhibition of tumor cell migration after *LINC01271* knockout (Figure 8E). In conclusion, knockout of *LINC01271* visibly suppressed the proliferation and migration of glioma cells, proving that LINC01271 positively regulates the progression of glioma and lncRNA LINC01271 is expected to become a new target for the treatment.

## DISCUSSION

The high fatality rate, ease of development, and propensity for metastasis make glioma a formidable threat to human health. It is of great clinical significance to search for prognostic biomarkers and immunotherapy targets for glioma. The present study utilized data from public databases TCGA and CGGA to collect glioma patients’ information, and identified 6 PD-L1-related lncRNAs for the construction of a risk score model. The findings indicated that glioma patients with a high-risk score exhibited significantly poorer prognosis and more malignant tumor pathological characteristics and microenvironment. Considering previous research results, we chose LINC01271 for further study separately. We revealed that the high LINC01271 expression level was relevant to poor glioma prognosis (especially LGG), aggressive clinicopathological features, and immunoinflammatory characteristics. Subsequent *in vitro* cell experiments have confirmed that knocking out LNC01271 effectively suppresses the proliferation and migration of glioma cells, indicating that LINC01271 holds great potential as a biomarker for glioma and as an immunotherapy target.

The PD-1 receptor is encoded by the *PDCD1* gene, while PD-L1 is encoded by the *CD274* gene; both are expressed on activated T cells. Many tumors use PD-L1/PD -1 signals to achieve immune escape [25]. The usage of ICIs to block PD-L1/PD-1 has made significant progress in a variety of tumors, including lung cancer. However, due to differences in the immune microenvironment and tumor cell immunogenicity, there is no encouraging progress in the treatment of glioblastoma with ICIs [26]. Currently ongoing research confirm that PD-L1 expression is associated with the glioma prognosis. Zhu et al. found that the expression of PD-L1 in gliomas tended to be dependent on tumor grade, with higher levels associated with shorter overall survival [25]. Previous studies have also demonstrated that PD-1 and PD-L1 expression are negative predictors of GBM prognosis [27]. Our research showed that patients with high-risk scores exhibited elevated expression levels of 5 immune checkpoints and displayed a greater propensity to respond favorably to anti-PD-1 treatment, which is consistent with previous studies. This may be related to the more malignant GBM patients, especially those with the mesenchymal subtype [28], as they tend to express higher levels of PD-L1 in their tumor cells.

The matrix components in the TIME influence the effect of PD-L1 expression on prognosis. Qian et al. found that the level of IFN-γ in mouse gliomas is positively correlated with the PD-L1 expression, and scores based on IFN-γ-induced genes can serve as supplementary prognostic indicators for anti-PD-1/PD-L1 therapy [29]. Moreover, PD-L1 can also be expressed in GBM extracellular vesicles, playing an immunosuppressive role through monocytes rather than T cells [30]. This suggests that the immunosuppressive signalling pathway involved in PD-L1 is complex.

The expression of CTLA-4, another immune checkpoint, competes with the costimulatory receptor CD28 for binding its ligands CD80 and CD86 [31], which also obviously increased after T cell activation. Reardon et al. revealed that the combination therapy targeting CTLA-4 and PD-1 cured 70% of glioma mice, higher than the cure rate of PD-1 inhibitor alone, and induced tumor-specific memory effect to prevent recurrence [32]. This demonstrated that CTLA-4 inhibitory therapy might also improve the prognosis of patients with glioma. Still, unfortunately, there is no significant difference in response to the anti-CTAL-4 treatment of the two groups in our experiment. The above research suggests that immune checkpoint suppression therapy for glioma may involve more internal mechanisms.

Our previous research found that the prognosis of LGG patients with epilepsy surrounded by a diverse immune microenvironment is different [33]. The interaction between immune cells and inflammatory factors within the microenvironment modulates tumor progression [34]. The microenvironment of glioma promotes angiogenesis and inflammation, leading to high permeability of the blood-brain barrier (BBB) and facilitating immune cell recruitment. Our study found that macrophages (M0, M1) were more highly expressed in the high-risk score group. Macrophages are traditionally divided into M1 and M2 subtypes: M1, which is pro-inflammatory and secretes inflammatory factors such as IL-6, IL-8, and TNF-α, while M2 releases anti-inflammatory factors such as IL-10 and TGF-β. MI macrophage has anti-tumor properties while M2 type positively regulates the growth and migration of tumor cells [35]. In previous studies, M2 macrophages have been found to promote tumor growth and are associated with shorter survival times [36]. M2 polarization of macrophages was also significantly correlated with PD-L1 high expression [36]. Our study showed that although M2 macrophages in different risk score groups had a high expression, there was no significant difference. This may be because lncRNA regulation of gliomas does not confine to the polarization of macrophages. The glioma microenvironment comprises non-tumor cells, such as infiltrating or resident immune cells, other glial cells, vascular cells, etc., particularly tumor-associated macrophages (TAMs) [37]. TAM promotes the development of tumors through activating inflammatory response, destructing the BBB [38, 39], regulating glioma metabolism [40], and promoting immunosuppression [41]. GBM often resists ICIs treatment; the therapy that targets PD-L1 expression may overcome resistance to immune checkpoint blockade [42]. In addition to TAMs, T cells occupy most of the lymphocytes. Han et al. found that the abundance of CD8^+^ T cells was negatively correlated with tumor grade in gliomas, which is contrary to CD4^+^ T cells. The high presence of CD4^+^ T cells and low presence of CD8^+^ T cells represent poor patient prognosis [43]. As a subset of CD4^+^ T cells, Treg has high diversity and plasticity, and plays a vital role in immune tolerance through the CTLA-4 and PD-L1 pathways [44]. However, in this study, both CD8^+^ T cells and Treg cells existed more in the high-risk group, while the latter group had higher naive CD4^+^ T cells. The observed phenomenon could potentially be attributed to the stratification based on the risk score of lncRNAs associated with PD-L1 and the complexity of immunity.

In terms of inflammatory characteristics, we found that irrespective of whether it is based on the clinical model comprising 6 PD-L1-related lncRNAs or solely LINC01271 expression, the high-scoring group exhibited heightened expression levels of STAT1, MHC, HCK, LCK, and Interferon molecules. Studies have shown that high expression of signal transducer and activator of transcription 1 (STAT1) in brain gliomas is associated with poorer OS in patients [45]. In terms of mechanism, STAT1 may mediate epithelial-mesenchymal transition through the wnt/β-catenin signalling pathway to increase glioblastoma growth and migration [46]. Moreover, STAT1 production decreased in LGG with IDH mutant, and the expression of cytotoxic T lymphocyte-related genes and IFN-γ inducible chemokines is reduced [47], which may partially explain that the prognosis of IDH mutant is better than wild-type patients. The immunogenicity of tumors can be determined by the expression of major histocompatibility complex (MHC)-I. Previous studies revealed that gliomas reduce T cells’ recognition and killing by downregulating MHC-I and antigen-processing machinery (APM) components, thus evading immune surveillance [48, 49]. However, TMZ increases MHC-I expression through the NF-κB pathway to achieve a therapeutic effect [50].

Recent studies have shown that gene mutations regulated by epigenetics have become a significant feature of glioma subtypes [51]. Previous studies have used 1p/19q chromosomal codeletion, mutations of IDH1/2, and MGMT promoter methylation as three molecular biomarkers to guide the classification and treatment decisions of glioma [52]. Verhaak et al. classified gliomas into neural (NE), proneural (PN), classical (CL), and mesenchymal (MES) based on the histological type [53]. Among them, patients in the PN subtype have the best median survival time; MES subtypes have the worst prognosis with the most apparent inflammation features (genes of TNF and NF-κB pathways are significantly up-regulated) [54]. MGMT promoter unmethylated makes tumor cells less likely to benefit from DNA alkylation agents such as temozolomide or nitrosourea, and such patients have a shorter median survival [55]. 1p/19q non-codel, IDH wild type, MGMT promotor unmethylated, plus CL and MES subtype and higher WHO grade are associated with poor outcomes of glioma [56]. Currently, molecular biology markers are recommended for routine examinations in the pathological diagnosis of gliomas according to guidelines, and targeted therapies based on genetic and immune characteristics are also being studied with enthusiasm. This study found that whether it was based on the risk-scoring model of 6 PD-L1-related lncRNAs or grouped only by LINC01271 expression level, the results showed the same results as previous studies, which illustrated the rationality and feasibility of the model.

As a non-coding regulatory RNA, lncRNA has been proven to be a robust biomarker in many diseases in recent years. The present study selected 6 PD-L1-related lncRNAs, based on which a clinical model was developed with improved efficiency in predicting glioma prognosis. Diermeier’s team screened a series of lncRNAs that were overexpressed in mouse breast tumors compared to normal mammary epithelial cells and named them Mammary Tumor-associated RNA (MaTAR) [24]. Among them, MaTAR25 functions by regulating the expression of the gene Tensin1 via purine-rich element binding protein B (PURB), affecting tumor proliferation, migration and invasion. Subsequently, they identified LINC01271 as a direct homolog of MaTAR25 in humans and demonstrated that increased LINC01271 expression was associated with higher levels of malignancy [23]. Here, we show that LINC01271 expression in human gliomas is also associated with poor prognosis, which may be a new therapeutic target.

Unfortunately, few studies on the other 5 lncRNAs. Wang et al. found that MIR4500HG, among five immune-related genes, was a predictor of poor prognosis and early recurrence in stage Ia-b non-small cell lung cancer (NSCLC) [34]. Liang et al. reported the association of AC011891.3 with poor prognosis of prostate adenocarcinoma, which may serve as a potential immunotherapeutic target [57]. LINC02594, also known as CTC-501O10.1, exhibits up-regulation in the plasma of patients diagnosed with gastric cancer and thus holds potential as a biomarker for detecting this malignancy [58]. AL357055.3(lnc-SLC16A1-2:1) has not been reported, but its homologous protein SLC16A1 is a confirmed poor prognosis marker in various malignant tumors, possibly due to its involvement in glycolysis and glucose metabolism synthesis [59, 60]. AL355974.3, also referred to as lnc-COL4A1-7:1 in the human non-coding RNA library, lacks functional characterization in existing literature. There is still much to be unveiled regarding the mechanism by which lncRNA contributes to tumor progression.

GSEA analysis showed that LINC01271 is associated with the enrichment of hallmark gene sets, such as APOPTOSIS, COAGULATION, COMPLEMENT and E2F TARGETS. This suggests that LINC01271 may play a role in tumors through immune pathways. E2F transcription factors (E2Fs) are a set of genes encoding transcription factor families. They participate in the core transcription that drives the cell cycle process through the cyclin-dependent kinase (CDK)- retinoblastoma (RB)-E2F axis [61]. E2Fs have been implicated in the progression of breast cancer [62], lung cancer [63], chromophobe renal cell carcinoma [64], among others, however, the specific contributions of individual family members remain unclear. Liao et al. found that E2Fs expression increased in the human brain and central nervous system tumors and was associated with poorer OS of LGG and GBM and increased immune cell infiltration in the two tumors, proving that E2Fs may become promising prognostic biomarkers and immunotherapy targets of gliomas [65], which may also have a connection with the mechanism of LINC01271 in glioma.

This study has the following limitations: the sample data only comes from the TCGA and CGGA databases, and the sample size is not large, which may not objectively reflect the characteristics of glioma. Only bioinformatics analysis and cell experiments *in vitro* were obtained to verify the effect of LINC01271 on glioma without exploring specific downstream mechanisms. We will improve the *in vitro* experiment and in-depth study of possible molecular mechanisms in the next step.

## CONCLUSIONS

This study established a glioma risk score model based on 6 PD-L1- related lncRNAs from an immunological perspective, which can effectively reflect the clinical characteristics, prognosis, pathological features, immunoinflammatory microenvironment and immunotherapy reactivity of glioma patients. The application value of LINC01271 in predicting the prognosis and treatment of glioma is evident. In conclusion, we have identified a potential risk stratification approach and biomarker that could offer early prognosis prediction and personalized immunotherapy guidance for glioma, thereby contributing to the optimization of secondary and tertiary prevention.

## MATERIALS AND METHODS

### Data collection

RNA transcript data (Workflow Type: HTSeq-Counts) with glioma were respectively downloaded from the TCGA database and CGGA database [66], along with baseline clinical characteristics (age, gender, WHO grade, cancer type and subtype) and clinical pathological features (isocitrate dehydrogenase (IDH) Mutation status, 1p/19q gene status, DNA methylation of O6-methylguanine-DNA-methyltransferase (MGMT) and somatic mutation). The data was trained in the TCGA database and validated in the CGGA database.

### Immune and inflammatory characteristics

The CIBERSORT deconvolution algorithm was performed to explore the immune microenvironment [67]. Inflammation characteristics were reflected by gene set variation analysis (GSVA) with the expression of 7 kinds of inflammation-related molecules, including MHC-I, MHC-II, STAT1, LCK, HCK, IgG and interferon [68]. Beyond that, draw correlograms and heatmaps respectively by the “corrgram” and the “pheatmap” R packages to confirm the correlation.

### Risk score model and consensus clustering analysis

According to the expression of 6 PD-L1-related lncRNAs in samples, the risk score model was established with the formula: $risk\;score=\sum_{i=1}^{n}\left(\text{Gene}\;\text{exp}\;r_{i}\times \beta_{i}\right)$. Patients were divided into high/low-risk score groups with the median risk score. The R package “rms” was adopted to construct a nomogram for a new risk model; then we evaluated the efficiency of which with the AUC value of the receiver operating curve (ROC). Consensus Clustering Analysis was taken on to further verify the rationality of the risk score model [69].

### Functional enrichment analysis

To further analyze the function of signature lncRNAs, gene ontology (GO) analysis was applied to study the essential biological functions of signature lncRNAs that are conspicuously related to risk scores. GSEA determined the immune pathways related to signature lncRNAs based on the hallmark gene set [70, 71].

### Response to immunotherapy

The response of glioma to ICIs was predicted by the tumor immune dysfunction and exclusion (TIDE) algorithm [72]. The R package “pRRophetic” was adopted to predict the sensitivity to temozolomide (TMZ) treatment with data from the Genomics of Drug Sensitivity in Cancer (GDSC) database [73].

### Cell transfection

Human glioma cells U251 and human glioblastoma cells U87 were purchased from the iCell Bioscience Inc. (Shanghai, China) company. According to the transcription of the LINC01271 gene, three kinds of siRNA (si-LINC01271-334, si-LINC01271-1196, si-LINC01271-1564) were designed to silence LINC01271 gene expression in cell transfection experiments using Lip2000. Examine the silencing efficiency through quantitative real-time PCR (PCR primer information for LINC01271 and the synthesis information for related siRNA are shown in Supplementary Tables 2, 3) [74].

### Cell counting kit-8 assay

Cells were inoculated into a 96-well plate at a density of 5×10^3^ cells per well overnight and were incubated for 4 hours after adding 30ul CCK-8 solution. A spectrophotometer (Bio-Tek) measured the absorbance at 450nm to evaluate cell proliferation.

### EdU image kit

Cells were incubated in 50uM 5-Ethynyl-2’-deoxyuridine (EdU) medium for 2 hours, fixed with 4% paraformaldehyde, and permeated with 0.5% TritonX-100 penetrant. Apollo and DAPI were used for staining, imageJ merge images were used to determine cell proliferation, and quantitative analysis was performed by flow cytometry.

### Colony formation and cell migration assay

For clone formation assay, the treated cells were inoculated in 6-well plates (200 cells per well) for 2-3 weeks at 37° C, with 5% CO_2_ and saturated humidity, rinsed twice with phosphate buffered saline (PBS), fixed with 4% paraformaldehyde for 15min, stained with crystal violet staining solution after fixed and counted colony. To evaluate cell migration ability, the cells were added to the Transwell upper chamber by 2 x 10^6^ cells/ml and suspended in 0.2ml serum-free medium for 48h. The migrating cells were fixed, stained, and counted after incubation.

### Statistical analysis

All statistical analysis was accomplished with R software (version 3.5.3). The Student’s t-test, Wilcoxon rank sum test and Pearson’s Chi-squared test were used for comparing differences in continuous or categorical variables between groups. The Least Absolute Shrinkage and Selection Operator (LASSO) analysis, and multivariate and univariate Cox regression analysis were performed to screen for variables of interest. P <0.05 was considered statistically significant. Survival analysis is carried out by the Kaplan-Meier method with the “survival” R package (version 3.6) and calculated OS, Disease-Specific Survival (DSS), and Progression-Free Interval (PFI) as prognostic indicators.

### Consent for publication

All authors give their consent to publish this manuscript.

### Availability of data

All data are available in public repositories, which are listed in the main context.

## Supplementary Material

Supplementary Figures

Supplementary Tables
