# Supplementary material for: PD-L1-related IncRNAs are associated with malignant characteristics and immune microenvironment in glioma
Source: Aging (Albany NY). 2023 Oct 12;15(19):10785–810. doi: 10.18632/aging.205120 (PMC10599717; doi:10.18632/aging.205120)
Supplement: Supplementary Figures [file aging-15-205120-s001.pdf]

SUPPLEMENTARY FIGURES

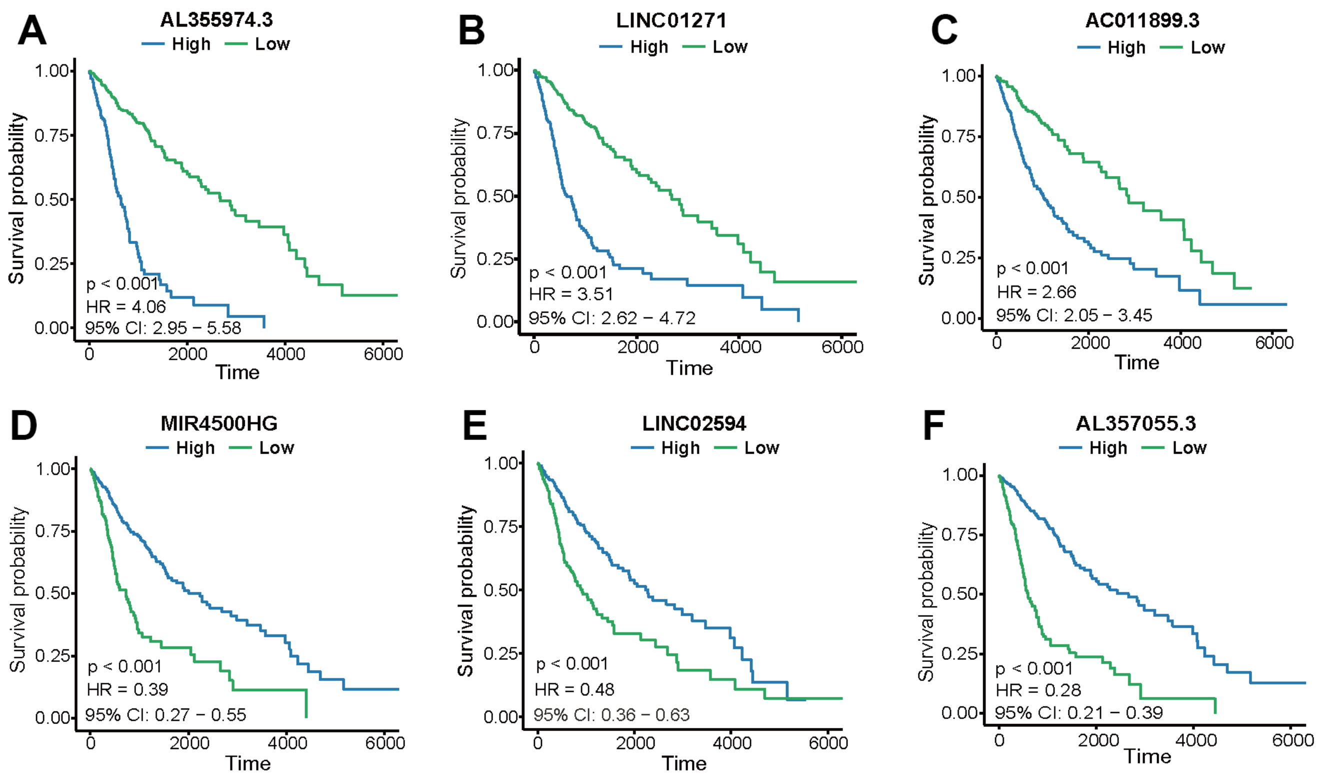

Supplementary Figure 1. The expression of PD-L1-related lncRNAs is associated with glioma patients' prognosis. (**A**–**F**) Survival analysis curves of AL355974.3 (**A**), LINC01271 (**B**), AC011899. 3 (**C**), MIR4500HG (**D**), LINC02594 (**E**), and L357055.3 (**F**) with high and low expression level.

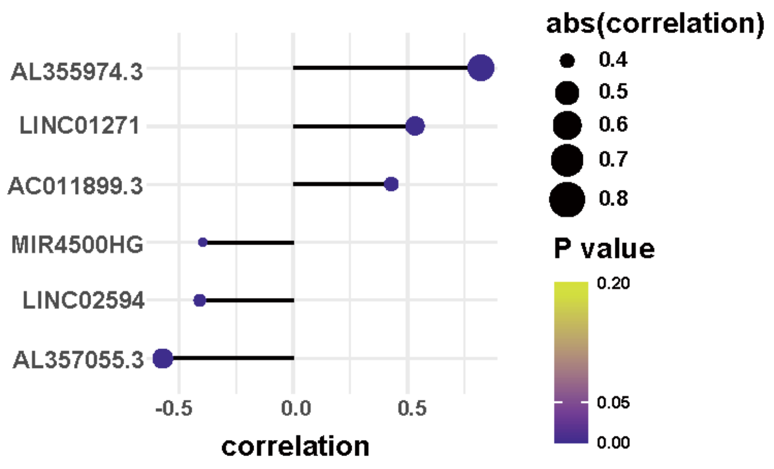

Supplementary Figure 2. Correlation between 6 PD-L1-related lncRNAs and risk score.

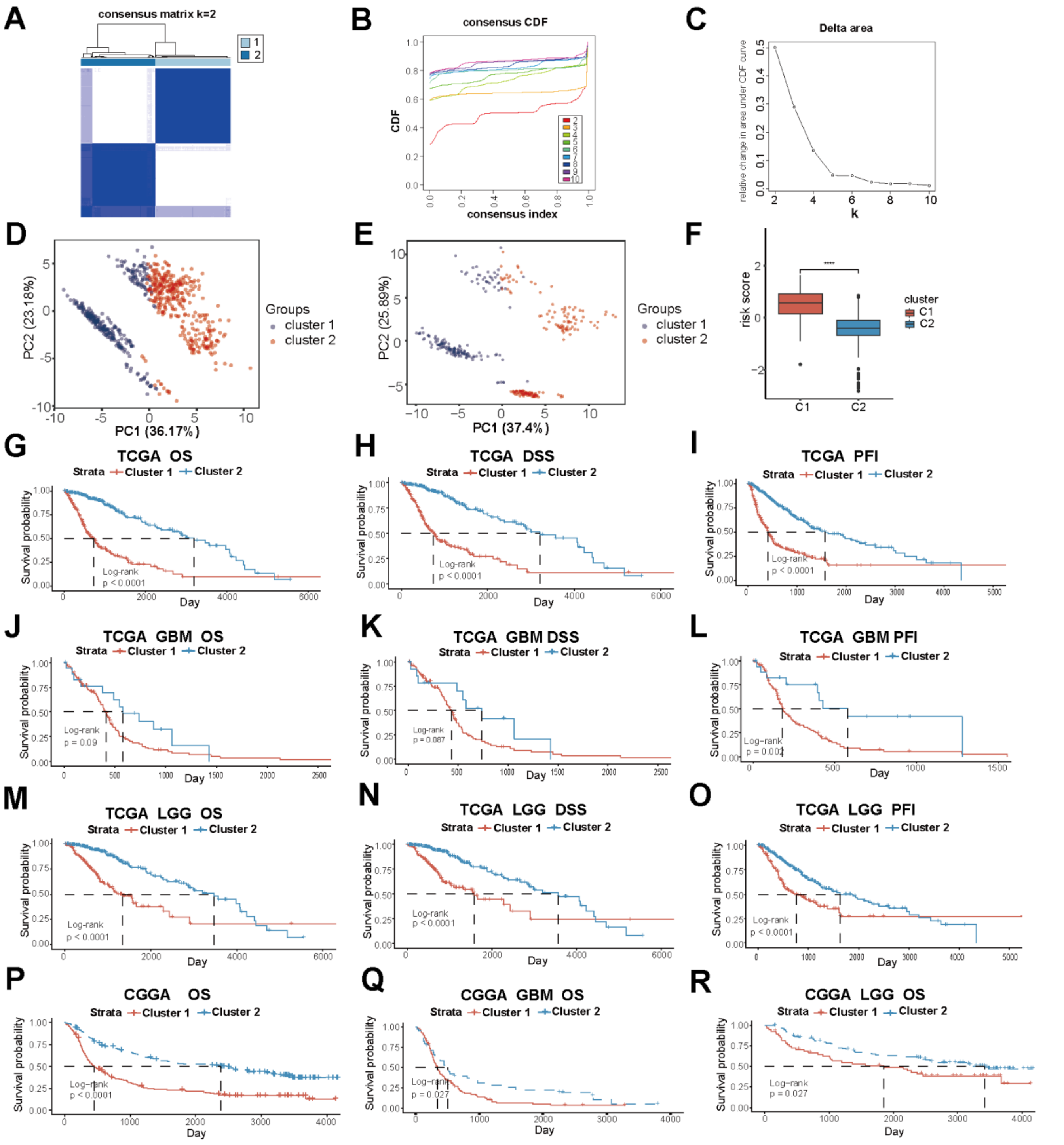

Supplementary Figure 3. Consensus cluster analysis reflects glioma patients' prognosis. (**A**–**C**) Consensus cluster analysis divided samples into two groups. (**D**, **E**) Principal component analysis. (**F**) Risk scores of two clusters. (**G**–**I**) OS, DSS, PFI in two clusters of all glioma patients in TCGA dataset. (**J**–**O**) OS, DSS, PFI of GBM and LGG subtype patients of two clusters in TCGA dataset. (**P**–**R**) OS of all patients, GBM and LGG patients in CGGA dataset of two clusters.

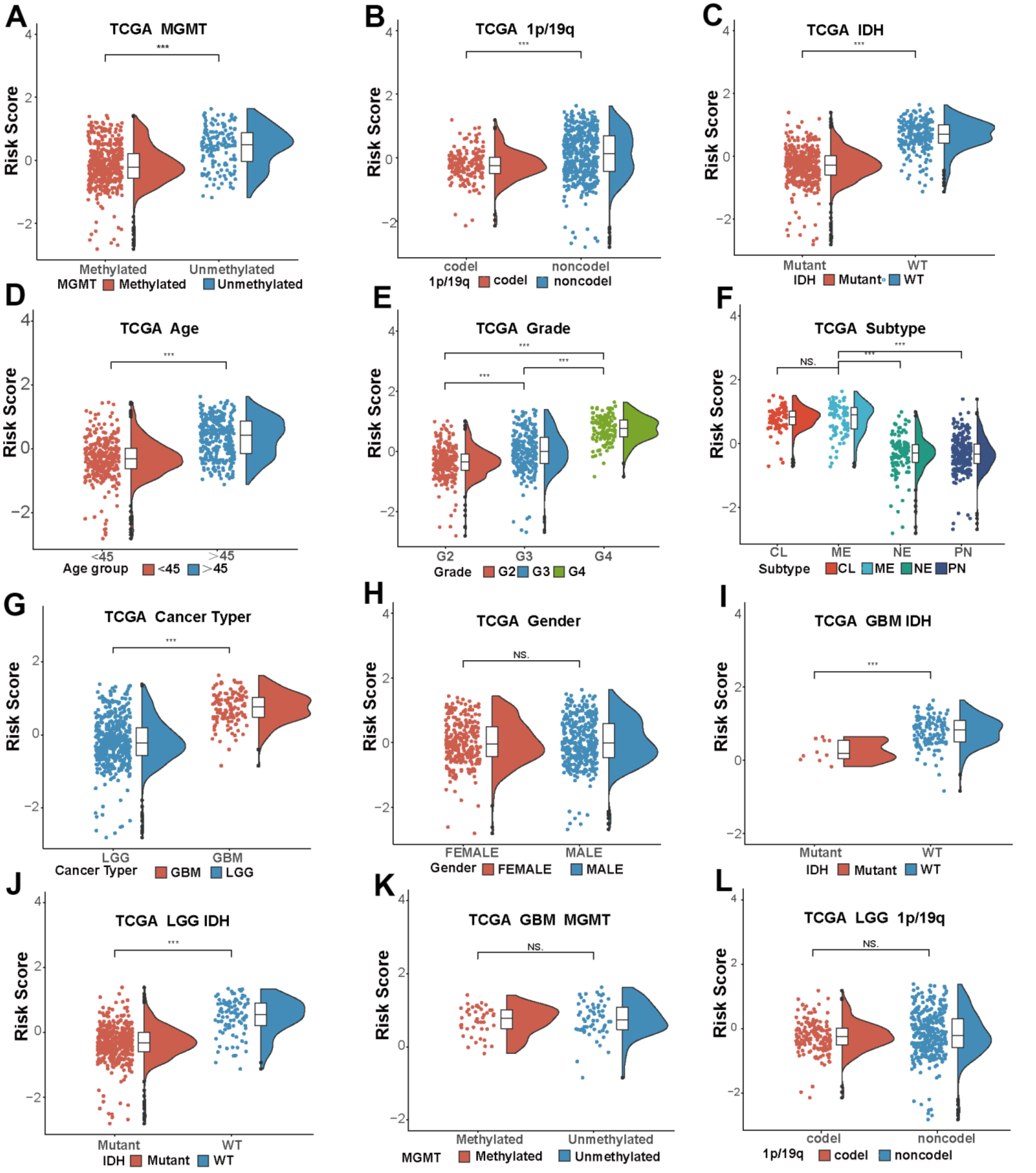

Supplementary Figure 4. Quantitative analysis of clinical and glioma pathological characteristics in different risk score groups in TCGA dataset. (**A**–**H**) Comparison of clinical and pathological features in all glioma patients. (**I**, **J**) IDH type of different risk score groups among GBM and LGG patients. (**K**) MGMT methylation status of different risk score groups among GBM patients. (**L**) 1p/19q co-deletion status of different risk score groups among LGG patients.

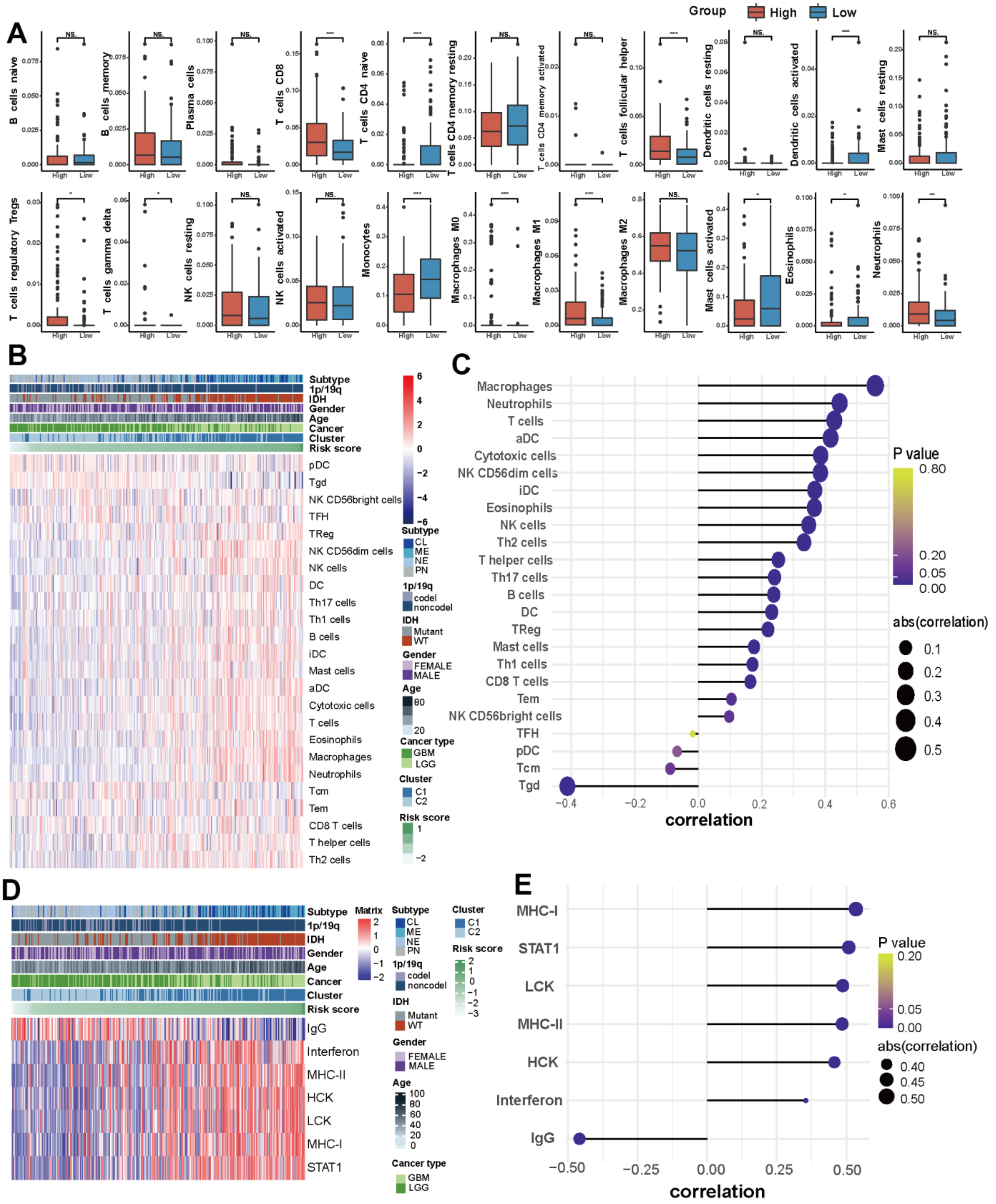

Supplementary Figure 5. Risk score reflects immune and inflammatory characteristics of glioma. (**A**) Quantitative analysis of the immune cells' expression in different risk groups in TCGA dataset. (**B**) Heatmap displaying the characteristics of immune cells panel in CGGA dataset. (**C**) The correlation between immune cell infiltration and risk score in CGGA dataset. (**D**) Heatmap displaying the characteristics of inflammation panel in CGGA dataset. (**E**) The correlation between inflammation characteristics and risk score in CGGA dataset.

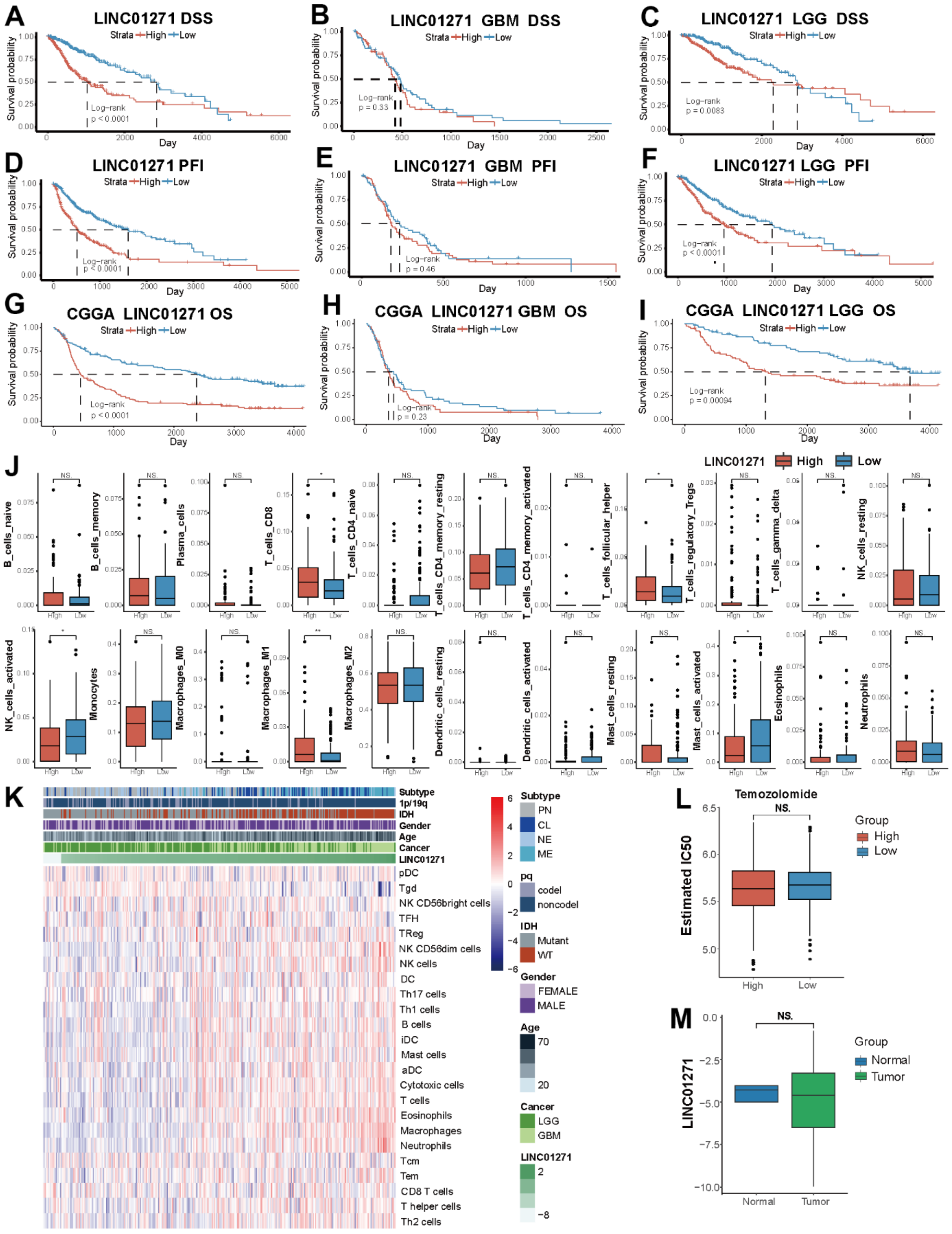

Supplementary Figure 6. Correlations between LINC01271 expression and the prognosis, immune and inflammatory features of glioma. (**A**–**F**) DSS and PFI among all glioma patients, GBM and LGG patients with different LINC01271 expression in TCGA dataset. (**G**–**I**) OS of all glioma patients, GBM and LGG patients with different LINC01271 expression in CGGA dataset. (**J**) Quantitative analysis of the expression of immune cells in different LINC01271 expression groups. (**K**) Heatmap displaying the characteristics of immune cells panel in CGGA dataset. (**L**) Sensitivity to TMZ treatment of patients with different LINC01271 expression. (**M**) LINC01271 expression in tumor and normal tissues in TCGA dataset.
