# Supplementary material for: PD-L1-related IncRNAs are associated with malignant characteristics and immune microenvironment in glioma
Source: Aging (Albany NY). 2023 Oct 12;15(19):10785–810. doi: 10.18632/aging.205120 (PMC10599717; doi:10.18632/aging.205120)
Supplement: Supplementary Tables [file aging-15-205120-s002.pdf]

SUPPLEMENTARY TABLES

Supplementary Table 1. Multivariate cox regression of PD-L1-related lncRNAs.

Characteristics	Hazard.Ratio	CI95	P.value
A1BG-AS1	1.1	0.95-1.28	0.198
AC009041.3	0.98	0.91-1.05	0.52
AC009955.1	1.03	0.96-1.12	0.393
AC011899.3	1.07	1-1.15	**0.038**
AC093726.2	1.06	0.9-1.24	0.492
AC131157.1	0.96	0.89-1.03	0.218
AC148476.1	1	0.95-1.07	0.897
ADAMTSL4-AS1	0.93	0.84-1.04	0.199
AL157702.2	1	0.95-1.05	0.97
AL355974.3	1.19	1.08-1.31	**0.001**
AL357055.3	0.78	0.65-0.93	**0.007**
AL442067.1	0.99	0.95-1.02	0.416
EPB41L4A-DT	1.12	0.94-1.35	0.212
LACTB2-AS1	1.09	0.99-1.19	0.071
LINC00887	1.06	1-1.12	0.051
LINC01271	1.07	1-1.15	**0.046**
LINC01504	1.06	0.97-1.15	0.208
LINC02594	0.95	0.91-0.99	**0.026**
LINC02612	0.99	0.94-1.04	0.684
MIR4500HG	0.93	0.88-0.98	**0.009**
SCAANT1	1.02	0.99-1.06	0.171
SNHG7	0.95	0.75-1.2	0.666
VLDLR-AS1	0.96	0.86-1.08	0.487

Supplementary Table 2. Primer sequences for LINC01271.

Name	Primer sequence (5'-3')
LINC01271-F	CGTCTCTGCACATTGTATGACC
LINC01271-R	CCTGGGATCTAGGGAGCTGAC

Supplementary Table 3. Synthesis information for siRNAs.

siRNA	Sense (5'-3')	Antisense (5'-3')
si-LINC01271-334	AGGAAAGACUGUAAAGAAUTT	AUUCUUUACAGUCUUUCCUTT
si-LINC01271-1196	GGACCAAUCACUAGAGCAATT	UUGCUCUAGUGAUUGGUCCTT
si-LINC01271-1564	GGACGGAAGUGGAGCAUCATT	UGAUGCUCCACUUCCGUCCTT
